# Prognostic factors and Doxorubicin involved in malignant progression of meningioma

**DOI:** 10.1038/s41598-023-28996-0

**Published:** 2023-04-06

**Authors:** Xulei Huo, Lairong Song, Ke Wang, Hongyi Wang, Da Li, Huan Li, Wei Wang, Yali Wang, Lei Chen, Zongmao Zhao, Liang Wang, Zhen Wu

**Affiliations:** 1 Department of Neurosurgery, Beijing Tiantan Hospital, Capital Medical University, Nansihuanxilu 119, Fengtai District, Beijing, 100070 China; 2 China National Clinical Research Center for Neurological Diseases, Beijing, China; 3 Department of Neurosurgery, Tianjin Fifth Center Hospital, Tianjin, China; 4 Department of Neuro-Oncology, Cancer Center, Beijing Tiantan Hospital, Capital Medical University, Beijing, China; 5 Department of Neurosurgery, The Second Hospital of Hebei Medical University, Shijiazhuang, China

**Keywords:** Cancer genetics, Cancer therapy, CNS cancer

## Abstract

Meningioma was the most primary intracranial tumor, but the molecular characteristics and the treatment of malignant meningioma were still unclear. Nine malignant progression-related genes based prognostic signatures were identified by transcriptome analysis between benign meningioma and malignant meningioma. The external dataset GEO136661 and quantitative Real-time Polymerase Chain Reaction were used to verify the prognostic factors. has-miR-3605-5p, hsa-miR-664b-5p, PNRC2, BTBD8, EXTL2, SLFN13, DGKD, NSD2, and BVES were closed with malignant progression. Moreover, Doxorubicin was identified by Connectivity Map website with the differential malignant progression-related genes. CCK-8 assay, Edu assay, wound healing assay, and trans-well experiment were used to reveal that Doxorubicin could inhibit proliferation, migration and invasion of IOMM-Lee Cells.

## Introduction

Meningioma accounts for 36.3% of primary intracranial neoplasms, and the occurrence rate was about 6–7 per 100,000 individuals^1^. It has fifteen kinds of subgroups, and the malignant meningioma includes atypical meningioma and the WHO 3 meningioma^2,3^. In addition, the meningioma with filtration of surrounding brain parenchyma was associated with poor outcome and recurrence^4–7^. Presently, the main treatment of meningioma was the surgery, and the adjuvant treatment (radiosurgery or fractionated external beam RT). And, several studies have shown that trabectedin, multi-kinase inhibitors, and bevacizumab could be used as the adjuvant treatment, but the results were unsatisfactory^8–10^.

Regardless of extraordinary efforts invested in the study of meningioma, the prognosis and treatment stayed unfavorable. Subsequently, further study on the underlying and important driven genes of meningioma and the potential compounds is desired. In the study, we focus on the prognostic factors and the potential compounds in the malignant progression of meningioma.

## Materials and methods

### Patient samples

Twenty meningioma patients were enrolled, and the patients had surgery between 2010 and 2020 at Beijing Tiantan Hospital. The clinical information included in the study were age, gender, pathology subgroup, location, progression-specific survival (PFS), and overall survival (OS). The clinical characteristics of the twenty patients (ten malignant patients and ten benign patients) was summarized in Table 1. The extent of resection was identified based on surgical records and the postoperative magnetic resonance imaging (MRI) within one month. The definition of gross total resection (GTR) was Simpson I (macroscopically complete tumor resection with removal of affected dura and underlying bone) and Simpson II (macroscopically complete tumor resection with coagulation of affected dura only)^11^. All patients did not receive chemotherapy or radiotherapy therapy. PFS was set the internal between the surgery time and the event that death or progression. OS was defined as the internal between the surgery time and the event that death or the follow-up.

**Table 1:** Clinical characteristics of patients.

Characteristics	Malignant (n = 10)	Benign (n = 10)	*p*-value
Sex (Male vs. Female)	30.0% vs. 70.0%	50.0% vs. 50.0%	0.21
Age (mean ± SD)	53.4 ± 11.58	52.9 ± 9.27	0.92
Location (supratentorial vs. cranial base)	60% vs. 40%	60% vs. 40%	1.00
Follow-up period (months) (mean ± SD)	93.1 ± 27.74	102.4 ± 23.65	0.43
Overall survival (months) (mean ± SD)	37.4 ± 18.15	102.4 ± 23.65	< 0.001
Progression-free survival (months) (mean ± SD)	15.9 ± 8.8	93.7 ± 34.2	< 0.001

All tissues were gathered immediately after the surgery, snap frozen in liquid nitrogen. Reviewers that blinded to the original diagnosis did histological diagnoses on formalin—fixed, paraffin-embedded hematoxylin and eosin sections. The Ethics Committee of Beijing Tiantan Hospital approved the study. All subjects signed written informed consent. All experiments were in accordance with Declaration of Helsinki of 2008.

### Whole-transcriptome library construction and sequencing

The library construction and sequencing steps was used as our previous study^12^. Two different libraries including lncRNA library and small RNA library were constructed and sequenced. For the lncRNA library, Trizol reagent kit (Invitrogen, Carlsbad, CA, USA) was used to extract the total RNA according to the manufacturer’s protocol. Agilent 2100 Bioanalyzer (Agilent Technologies, Palo Alto, CA, USA) and RNase free agarose gel electrophoresis were used to assess RNA quality. rRNAs were eliminated from the total RNA. The enriched mRNAs and ncRNAs were cut into fragments and converse transcribed into cDNA. DNA polymerase I, RNase H, dNTP (dUTP instead of dTTP) and buffer were used to synthesize second-strand cDNA. Next, QiaQuick PCR extraction kit (Qiagen, Venlo, The Netherlands) was utilized to purify the cDNA items. In addition, the items were end repaired, poly(A) added, and was ligated to Illumina adapters. Lastly, the second strand cDNA chain was removed by Uracil-N-Glycosylase. The final items were screened, magnified, and sequenced with the Illumina HiSeq 4000 of Gene Denovo Biotechnology Co. (Guangzhou, China).

Library was prepared for small RNA sequencing: after total RNA was extracted by trizol reagent kit (Invitrogen, Carlsbad, CA, USA), the RNA size range of 18–30nt were enriched by polyacrylamide gel electrophoresis. Then, 36-44nt RNAs with 3’ adapters were enriched. And, the RNAs ligated the 5’ adapters by PCR amplification. Final, 140–160 bp size items were enriched to produce the cDNA library. Illumina HiSeq 2500 by Gene Denovo Biotechnology Co. (Guangzhou, China) was utilized to sequence.

The adapter (lncRNA sequencing data) was removed from raw reads by fastp^13^ (version 0.20.1) to obtain high quality clean reads. The rRNA that removed from the sequence was mapped by Bowtie2 (version 2.4.4)^14^. And the follow steps were conducted as the published study^15^. The clean data were mapped to reference genome Human GRCh38 by hisat2 (version 2.2.1)^16^. And, for miRNA sequencing data, raw reads (miRNA sequencing data) removed the 3ʹ adapter, and the reads of out-range length or the low-quality reads were removed by cutadapt (version 3.4) to acquire high quality clean reads^17^. The ncRNA (snRNA, rRNA, and scRNA etc.) was collected from the Rfam 14 database and ensemble database and was removed from the clean data by blast (version 2.12.0)^18–20^. Final, clean data was mapped to reference genome hg38 by mirdeep2.pl of miRDeep2^21^. The BioProject number of the source data is PRJNA772033 depositing as FASTQ format.

### Identification of malignant progression-related genes

StringTie (version 2.1.7) and Python script (prepDE.py) were used to collect count results for each gene^15^. The count results of known miRNAs (miRbase database) were obtained by the quantifier.pl of miRDeep2^21,22^. Genes with Benjamini & Hochberg adjusted *P*-Value < 0.05 and | log FC (fold change) |> 1.0 were identified as differential malignant progression-related genes (MPRGs) by limma-voom pipeline^23–25^. Hierarchical clustering was used to display the MPRGs with “ComplexHeatmap” R package (version 3.15)^26^.

Weighted gene correlation network analysis (WGCNA)^27^ was utilized to reveal gene regulatory networks based on the differentially expressed mRNAs. Person’s connection coefficients were used to confirm a similitude framework (Sij) in a couple way and the soft threshold was set as 6. The matrix was trained into an adjacency network (aij) with aij = Power (Sij, β) ≡ |Sij| β. Network connectivity was estimated by the topological overlap matrix (TOM) and the corresponding dissimilarity (1-TOM) was calculated. Average linkage hierarchical clustering was conducted for the dendrogram dependent on the topological overlap of the association features. In addition, the network modules were decided by the dynamic tree cut with the least group size of 50.

### Functional enrichment analysis

Gene Ontology (GO) Biological Process term and Kyoto Encyclopedia of Genes, Genomes (KEGG) pathway analysis^28^, and gene set enrichment analysis (GSEA) were performed using “clusterProfiler” package^29^ based on the mRNA. The gene sets of h.all.v7.4.entrez.gmt (cancer hallmarks) and c7.all.v7.4.entrez.gmt (Immunologic signatures) were selected for cancer hallmarks and immunological hallmarks analysis in GSEA analysis.

### Identification of malignant progression-related genes based prognostic signatures

In order to make the results more confidential, we excluded RNAs that did not have the mutual interaction for the identification of prognostic signatures. The mRNA and miRNA pairs were generated by the multiMiR package^30^. miRNA target prediction was performed by two experimentally validated databases: miRTarBase database (https://mirtarbase.cuhk.edu.cn/~miRTarBase/miRTarBase_2022/php/index.php) and TarBase 8.0 (http://www.microrna.gr/tarbase)^31,32^. To improve prediction accuracy, the overlap of the predicted results from the two databases and the differential expressed mRNAs was considered to represent the predicted target mRNAs. In addition, lncRNA-miRNA pair was obtained from Starbase website (https://starbase.sysu.edu.cn/index.php)^33,34^. Similar with the miRNA-mRNA interaction, the overlap of the predicted results from the database and the differential expressed lncRNAs was considered to represent the predicted target lncRNAs. Lastly, the final mRNAs, miRNAs, and lncRNAs were used to create prognostic matrix.

Then, we performed univariate Cox regression analyses to determine the OS-related MPRGs (*P*-value < 0.01). Next, the LASSO (least absolute shrinkage and selection operator algorithm) analysis and multivariate Cox regression analysis were adopted to avoid overfitting and ultimately to screen out the significant OS-related MPRGs^35^. Finally, nine prognostic signatures were constructed for predicting the OS by the expression values of the MPRGs and the regression coefficients in the multivariate Cox regression analysis. The specific formula was follows:

$$ {\text{risk\,score}} = \sum (\beta_{n} \times {\text{ expression\,of\,gene}}_{n} {) } $$

Each patient got their risk score according to the formula, and they were divided into high-risk group and low-risk group according to the largest difference between true positive and false positive. The time-dependent receiver operating characteristic (ROC) curves and area under the curve (AUC) value were adopted to evaluate the predictive power of the MPRGs-based prognostic signatures. And, the expression of the nine MPRGs-based prognostic factors was visualised by “ComplexHeatmap” R package (version 3.15)^26^ in the malignant group and benign group.

### Validation of malignant progression-related genes based prognostic signatures

External validation is important for the prognostic signature. Total 161 patients in the GEO136661 dataset^36^ with the clinical characteristic including the invasion and the WHO grade was selected for the validation of the prognostic signatures. After converted read count value into Fragments Per Kilobase of exon model per Million mapped fragments (FPKM), the difference of the prognostic signatures among WHO group and the patients with or without the invasion characteristic was assessed.

In addition, the quantitative Real-time Polymerase Chain Reaction (qRT-PCR) was also performed to verify the nine prognostic factors. Total RNA was extracted with RNAsimple Total RNA Kit (Tiangen, Beijing, China) and was quantified by NanoDrop ND-1000 spectrophotometer (Thermo-Scientific, Waltham, MA, USA). Primers for mRNAs and miRNAs were synthesized by Biomed (Beijing, China). For miRNA, Equal amounts of total RNA (1 µg) were reverse-transcribed using the miRcute Plus miRNA First-Strand cDNA Kit (Tiangen, Beijing, China) and the expression levels was quantified by using miRcute Plus miRNA qPCR Kit (Tiangen, Beijing, China). For mRNA, Equal amounts of total RNA (500 ng) were reverse-transcribed using the PrimeScript RT Master Mix (Takara, Beijing, China) and the expression levels was quantified by using TB Green Premix Ex Taq II (Takara, Beijing, China). In addition, qPCR was performed in duplicates on ABI 7500 real-time PCR system (Applied Biosystems, Forest City, California, USA) as the published study^37^. Relative fold changes between the expression of target genes were calculated by using the 2^−∆∆CT^ method. GAPDH and U6 were used as reference genes. Relative expression mRNA levels of hsa-miR-3605-5p, hsa-miR-664b-5p, PNRC2, BTBD8, EXTL2, SLFN13, DGKD, NSD2, and BVES were normalized to the mean of the DMSO tumor samples. The primers of RNA used are listed in Tables S1 and S2.

### Potential compounds analysis

Connectivity Map (CMap), a comprehensive online analysis tool (https://clue.io/) to investigate underlying interactions between genes and chemicals^38^, was used to identifie potential candidate compounds that related with malignant progression-related genes. We selected the up-regulated genes within the differentially expressed genes, which have mutual interaction, to inquiry the dataset. The query results were ranked with scores from − 100 to 100. The top 50 compounds with enrichment score ≤  − 99 was selected as relative compounds. “ComplexHeatmap” R package (version 3.15) was used to graph the result^26^.

### Cell culture and half maximal inhibitory concentration

The human meningioma cell line, IOMM-Lee, was used as the published study^37^. IOMM-Lee cells were cultured in Dulbecco's modified Eagle medium (Gibco [Life Technologies, Thermo Fisher Scientific, Rockford, Maryland, USA) with 10% fetal bovine serum (FBS; Gibco [Life Technologies, Thermo Fisher Scientific]) at 37 °C in a humidified environment with 5% carbon dioxide atmosphere.

IOMM-Lee cells were seeded into 96-well plates at a concentration of 8 × 10^3^ cells per well, with duplicates. After 4 h, cells were treated with Doxorubicin (Selleck, China), the topoisomerase inhibitor, in 12 concentrations ranging from 1 to 400 nM and incubated for 48 h. Dimethyl sulfoxide (DMSO, Sigma-Aldrich, St. Louis, MO, USA) was used as the negative control group. Then, medium was removed, and cells were incubated with 100 µL medium with 10% Cell Counting Kit-8 (CCK-8, Dojindo Molecular Technologies, Inc., Kumamoto, Japan). Absorbance was measured at 450 nm with the Tecan Spark Microplate Reader (Tecan Group AG, Mannedorf, Switzerland) after 2.5 h. IC_50_ values were calculated by nonlinear regression in GraphPad Prism (version 9.0.0, GraphPad, San Diego, CA, USA). The cell viability rate (%) was calculated using Eq.: (A_s_ − A_b_)∕(A_c_ − A_b_) × 100%.

### Cell proliferation curve

A total of 8 × 10^3^ cells of IOMM-Lee was seeded in 96 well plates with 200 µL medium. After 4 h, cells were treated with DMSO control or Doxorubicin at IC_50_. After at 4, 24, 48, 72, and 96 h, the medium was removed, and cells was incubated with 10% CCK-8 for 1 h. Absorbance was measured at 450 nm with the Tecan Spark Microplate Reader (Tecan Group AG, Mannedorf, Switzerland). OD values were calculated in GraphPad Prism (version 9.0.0, GraphPad, San Diego, CA, USA).

### EdU assay

A 5-ethynyl-20-deoxyuridine (EdU) assay kit (Solarbio, Beijing, China) was adopted to inquire the cell proliferation ability. Cells were seeded into 96-well plates with a density of 4 × 10^4^ cells each well. After 4 h, the cell monolayer was washed carefully with 100 μL PBS to remove cell debris and thereafter 1 mL fresh medium was added. Cells were treated with DMSO control or Doxorubicin at IC_50_. After 24 h, the cell monolayer was washed carefully with 100 μL PBS to remove cell debris and thereafter incubated with 50 μM EdU buffer at 37 °C for 2 h, fixed with 4% formaldehyde for 0.5 h and stained nuclei with Hoechst according to the manufacturer’s protocol. Then the results were visualized by a fluorescence microscope. Fluorescence cells were calculated by ImageJ (version2.3.0, National Institutes of Health, Bethesda, MD, USA).

### Wound healing assay

8 × 10^5^ cells were seeded in 6-well plates. After reaching 100% confluency, a straight gap was generated on the cell monolayer using a 100 µL pipette tip. Then, the cell monolayer was washed carefully with 1 mL PBS to remove cell debris and thereafter 2 mL 2% fresh medium was added. Doxorubicin was added at the concentration of IC_50_. DMSO (Sigma-Aldrich, St. Louis, MO, USA) was used as the negative control group. Images of the gap were taken at 0 and 24 h, respectively. Migrated areas of cells were calculated by ImageJ (version1.8.0, National Institutes of Health, Bethesda, MD, USA).

### Migration and invasion assays

Migration or invasion assays were performed using 24-well plates inserted by 8-μm pore size transwell filter insert (Costar 3422 [Corning, Corning, New York, USA]) with or without pre-coated diluted Matrigel (1:8) (Becton Dickinson and Co., Franklin Lakes, NJ, USA). 5 × 10^4^ IOMM-Lee cells with Serum-free medium were placed into the upper chamber, and 500 μL medium containing 30% FBS was added into the bottom chamber subsequently. Doxorubicin was added at the concentration of IC_50_. DMSO (Sigma-Aldrich, St. Louis, MO, USA) was used as the negative control group. After incubation in 37 °C for 24 h, cells on the underside of membrane were immobilized about 40 min with 4% Paraformaldehyde (Solarbio, Beijing, China) and stained about 20 min with 0.4% crystal violet (Solarbio, Beijing, China). Then penetrated cells were counted in five random fields under the microscope. Migrated areas of cells were calculated by ImageJ (version2.3.0, National Institutes of Health, Bethesda, MD, USA).

### Statistical analysis

All statistical analyses were performed with R (version 4.1.2, R: The R Project for Statistical Computing)or GraphPad Prism (version 9.0.0, GraphPad, San Diego, CA, USA) and a *p* value < 0.05 was considered statistically significant. All experiments were repeated 3 times to avoid contingency and ensure reliability of the results. Data are presented as the mean ± standard deviation. Fisher’s exact test was used to compare location, Gender, GTR, and Simpson grade between groups. Kaplan–Meier method was used and compared by log-rank tests. The differences between groups were analyzed using the independent sample t test or Mann–Whitney U test for the parametric and nonparametric variables, respectively. *p < 0.05; **p < 0.01; ***p < 0.001.

### Ethics approval and consent to participate

This study was approved by the Ethics Committee of Beijing Tiantan Hospital. The approved the study. All subjects signed written informed consent.

## Results

### Distinctive RNA expression between malignant progression group and benign progression group

**Figure 1:**
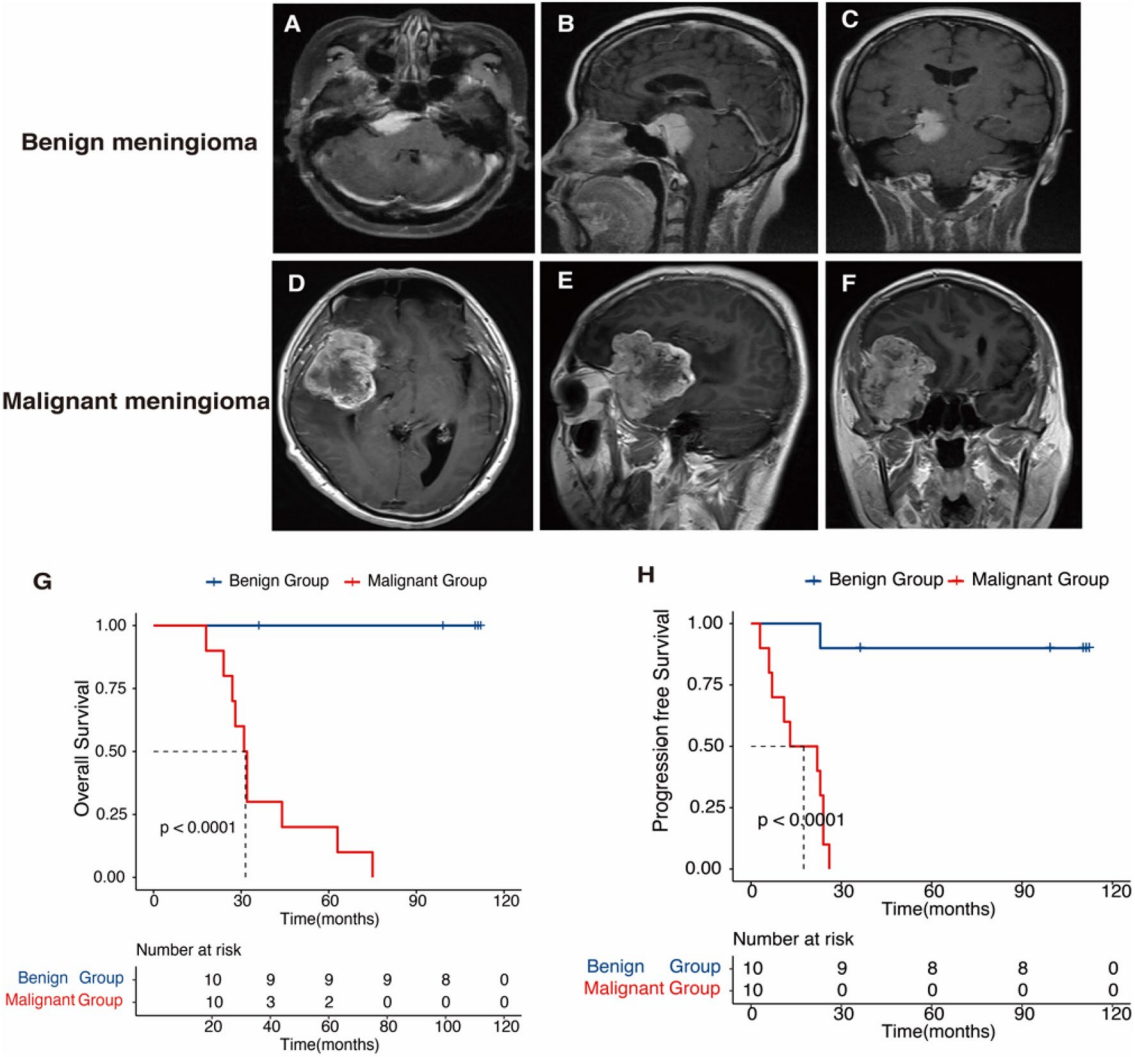
MRI of meningioma patients with benign progression and with malignant progression. (**A**, **B**, **C**) Same meningioma patient with WHO 1 pathology; (**D**, **E**, **F**) Same meningioma patient with WHO 3 pathology and the tumor penetrate the dura mater and invade into brain. Kaplan–Meier survival analysis of meningioma patients. (**G**) Meningioma patients with malignant progression have shorter overall survival time than patients with benign progression (*P* < 0.0001); (**H**) Meningioma patients with malignant progression have shorter progression-free survival time than patients with benign progression (*P* < 0.0001). Log-rank test was used as the method.

The benign meningiomas (10 cases) demonstrated expansive growth in magnetic resonance imaging (MRI). And, there was no serious brain invasion was found in surgical treatment (Fig. 1A,B,C). Contrary with that, malignant meningiomas (10 cases) demonstrated a lump from the neoplasm into cerebrum regions and dural invasion (Fig. 1D,E,F). The clinical information was summed up in Table 1. Age, gender, location, and follow-up from surgery were not statistically difference between the two groups. However, the distinction of the OS and progression-free survival (PFS) was essentially significant, with poorer outcome found in the malignant progression patients (Fig. 1G,H).

**Figure 2:**
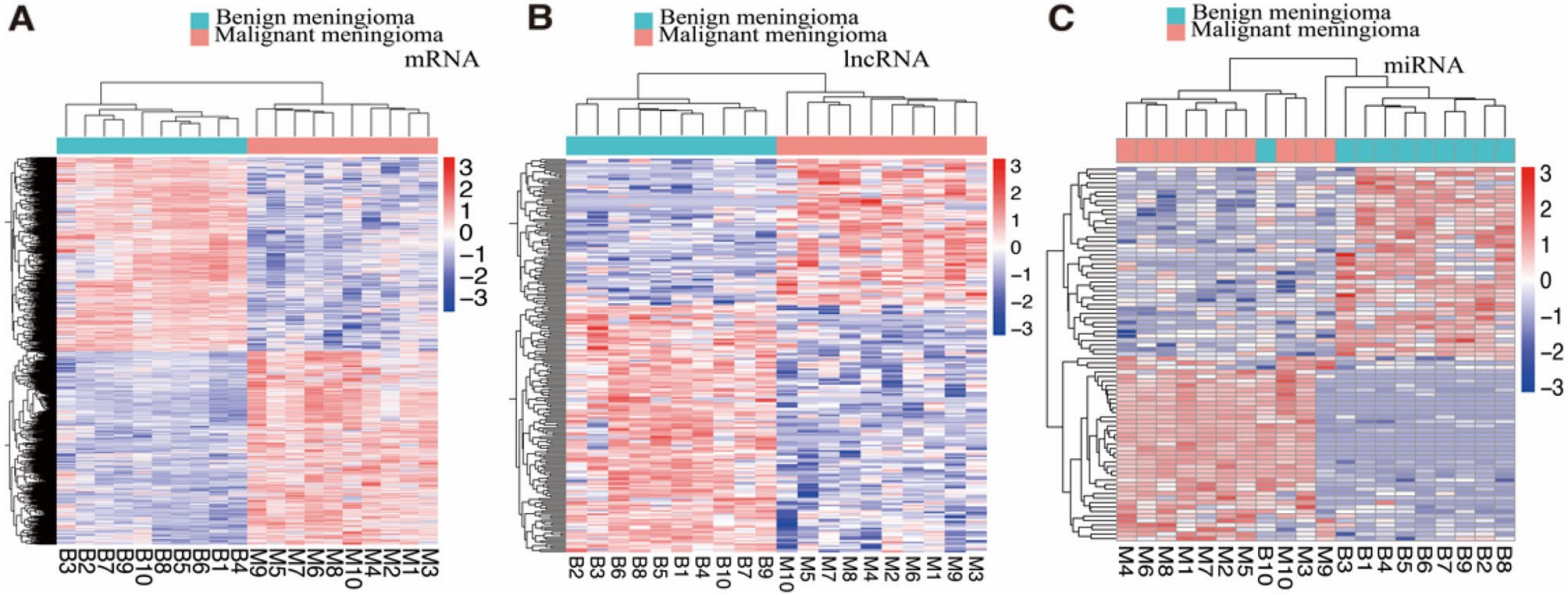
LncRNA, mRNA and miRNA expression profile changes between malignant group and benign group in meningioma. (**A**, **B**, **C**) Hierarchical clustering of all samples revealed the nonrandom partitioning of samples into two major group: one group containing ten malignant progression penetration samples and another group containing ten benign progression samples. Each column represents one sample and each row represents one lncRNA, mRNA and miRNA set. “ComplexHeatmap” R package (version 3.15) was used to graph the result.

The differential RNAs between the two groups were explored and the benign meningiomas were the control group. Totally, the 1253 differential mRNAs, 223 differential lncRNAs and 83 differential miRNAs (Fig. 2A,B,C) were identified with adjusted *p* values 0.05 and | log fold change |≥ 1. And, the WGCNA network was constructed to study the biological networks among the differentially expressed mRNAs. Finally, 1075 mRNAs were selected in co-expression turquoise module (R = 0.97, *p* < 0.0001) that was significantly correlation with the malignant meningioma group (Supplementary Fig. 1D).

**Figure 3:**
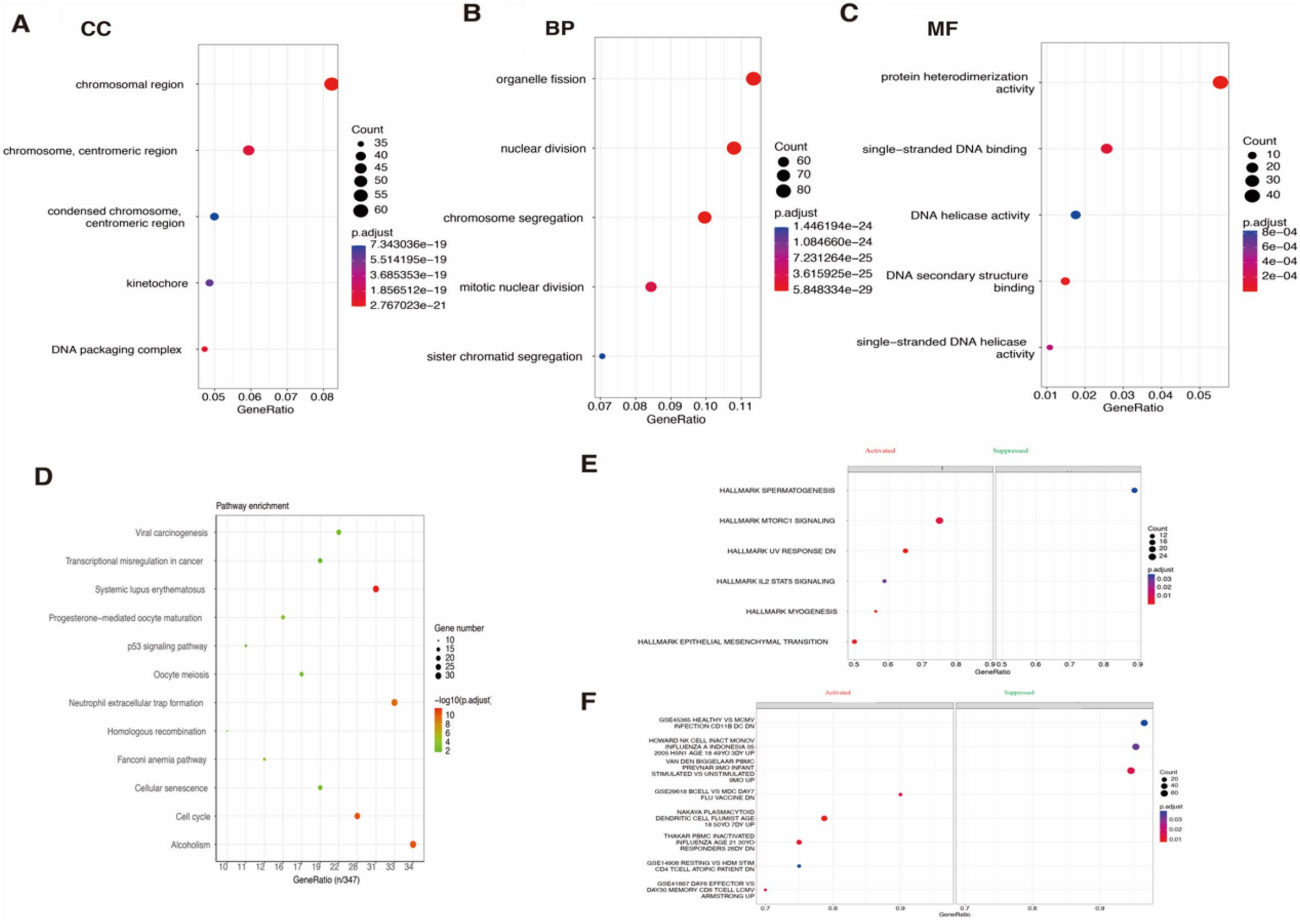
Functional pathway enrichment of malignant progression-related mRNAs in meningioma (**A**, **B**, **C**) Biological process, cellular component, and molecular function pathway enrichment (GO); (**D**) Kyoto Encyclopedia of Genes and Genomes pathway enrichment; (**E**, **F**) GSEA pathway enrichment of cancer markers and immunologic markers.

In addition, GO analysis revealed mRNAs were mainly enriched in the chromosome segregation, chromosomal region, and DNA helicase activity (Fig. 3A,B,C). And the KEGG analysis revealed the top five pathways were the cell cycle, Fanconi anemia pathway, alcoholism, neutrophil extracellular trap formation, systemic lupus erythematosus, and viral carcinogenesis (Fig. 3D). GSEA analysis revealed that five activated pathways and one suppressed pathway was enriched in cancer pathways (Fig. 3E). And, there are three activated pathways and five suppressed pathways were enriched in the immune processes (Fig. 3F).

### Identification of malignant progression-related genes based prognostic signatures

**Figure 4:**
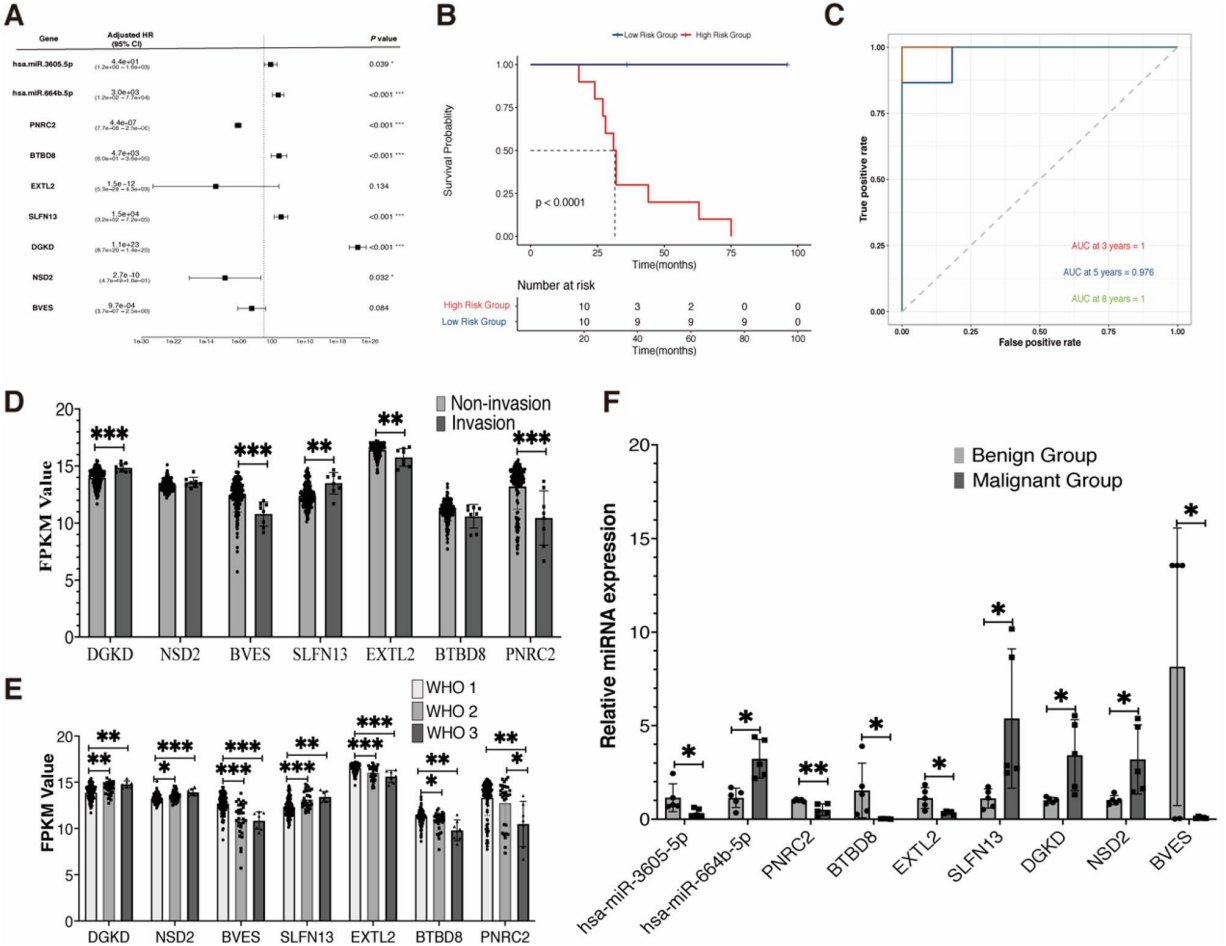
Identification and validation of nine MPRGs-based prognostic factors. (**A**) Forest plot of the impact of MPRGs-based prognostic factors on overall survival (OS); (**B**) Kaplan–Meier OS curve of high- and low-risk groups based on the risk score in the group; (**C**) Time-dependent ROC curves in our cohort; (**D**, **E**) The expression profile of seven MPRGs-based mRNAs between invasion group samples and no invasion group or among WHO grades samples in 157 meningioma tumors from the GEO136661 dataset. (**F**) Expression level of the nine MPRGs-based prognostic factors between malignant group samples and benign group samples were analyzed using qRT-PCR, U6 and GAPDH was set as internal control. Error bars represent the standard errors of independent samples. *P < 0.05, **P < 0.01, ***P < 0.001.

After excluding RNAs without mutual correction, 958 genes were identified. Univariate Cox regression analysis identified 453 genes with prognostic significance (*P* < 0.01). After LASSO regression analysis and stepwise multivariate Cox regression analysis, nine genes (hsa-miR-3605-5p, hsa-miR-664b-5p, PNRC2, BTBD8, EXTL2, SLFN13, DGKD, NSD2, and BVES) were identified to construct the optimal prognostic model (Fig. 4A). The risk score formula based on the gene expression levels and coefficients of these nine MPRGs based signatures was calculated as follows: $\mathrm{Risk score }= -3.791\times \mathrm{ miR}-3605-5\mathrm{p }+ 8.018 \times \mathrm{ miR}-664\mathrm{b}-5\mathrm{p }- 14.64 \times \mathrm{PNRC}2+ 8.447 \times \mathrm{ BTBD}8 - 27.22 \times \mathrm{ EXTL}2 + 9.63 \times \mathrm{ SLFN}13 + 53.04 \times \mathrm{DGKD }- 22.02 \times \mathrm{ NSD}2 - 6.938 \times \mathrm{ BVES}$. The expression level of miR-664b-5p, SLFN13, DGKD, and NSD2 was correlated with high risk while the expression level of miR-3605-5p, PNRC2, BTBD8, EXTL2, and BVES were correlated with low risk (supplementary Figs. 2, 3).

Kaplan–Meier survival analyses were conducted in the cohort to evaluate the predictive capability of the MPRGs based prognostic signatures. The risk score was calculated for each patient, and the patient was divided into high risk group (n = 10) and low risk group (n = 10). There were distinct survival outcomes between the two groups (*P* < 0.0001, Fig. 4B). The 8-year region under the curve (AUC), 5-year AUC, and 3-year AUC were 1, 0.976, and 1 (Fig. 4C).

### Validation of malignant progression-related genes based prognostic factors

To validate the prognostic factors, we analyzed the expression levels in the external GEO136661 dataset (Fig. 4D,E). DGKD (*P* < 0.001) and SLFN13 (*P* < 0.01) expressed higher in the invasion group while BVES (*P* < 0.001), PNRC2 (*P* < 0.001), and EXTL2 (*P* < 0.01) expressed higher in the contrast group. In addition, BVES (*P* < 0.001), EXTL2 (*P* < 0.001), PNRC2 (*P* < 0.05), and BTBD8 (*P* < 0.05) expressed higher in the low WHO grade group than the contrast group while DGKD (*P* < 0.01), NSD2 (*P* < 0.05), and SLFN13 (*P* < 0.001) expressed higher in high WHO group. Moreover, we also analyzed the expression levels in 10 meningioma tumor tissues (5 malignant meningiomas and 5 benign meningiomas) by qRT-PCR. The results were consistent with the bioinformation results (Fig. 4F).

### Doxorubicin inhibits proliferation, migration and invasion of IOMM-Lee cells

**Figure 5:**
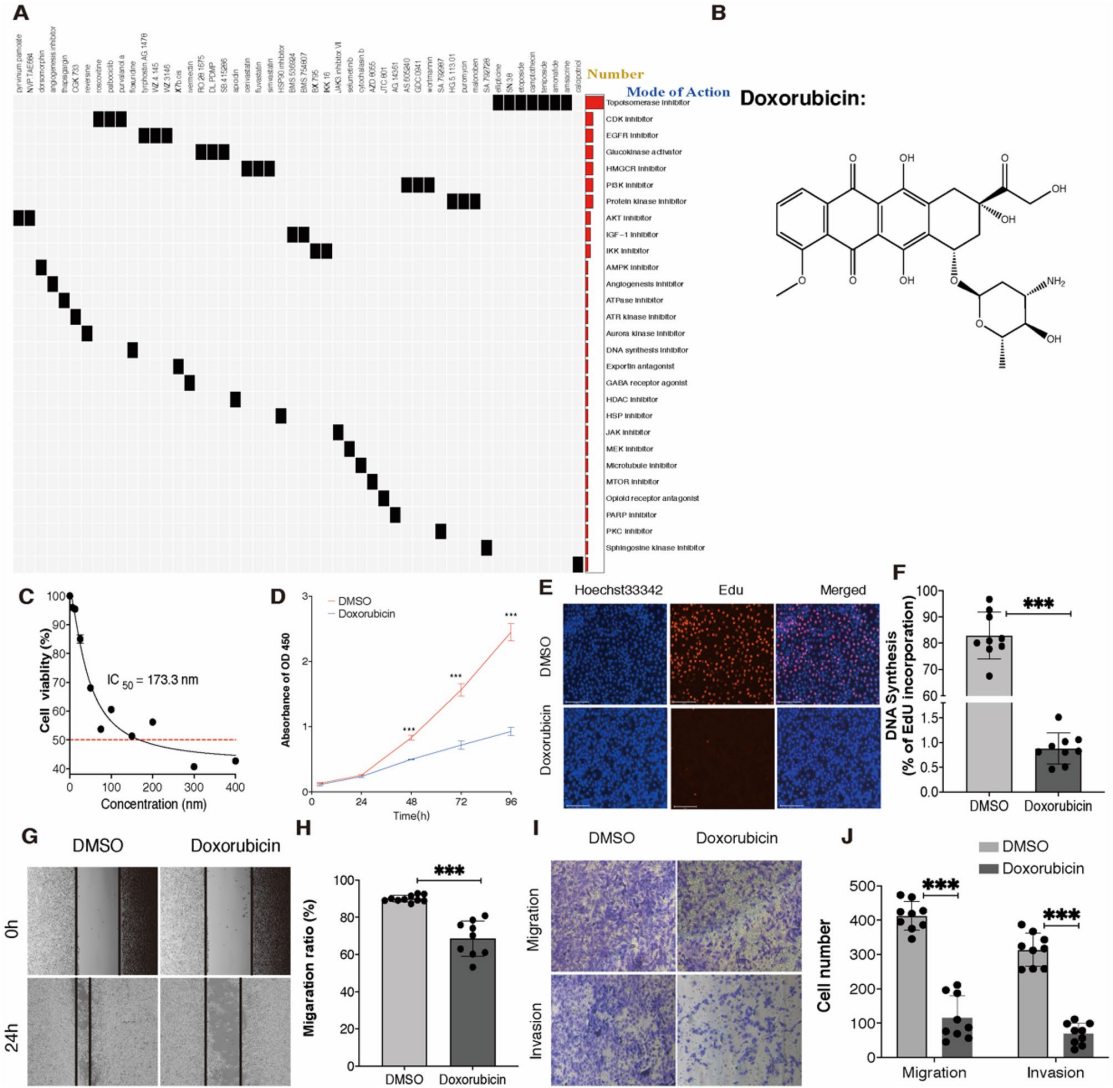
Doxorubicin inhibits proliferation, migration and invasion of meningioma. (**A**) Correlation of compound with differently expressed mRNA: Heatmap showing compounds (top) from the CMap shares mechanisms of action and the mechanisms of action was sorted by descending number of compounds (right); “ComplexHeatmap” R package (version 3.15) was used to graph the result. (**B**) The molecular structure of Doxorubicin; (**C**) The dose-curve of Doxorubicin in IOMM-Lee cell line; (**D**) CCK-8 assay of the cell proliferation ability in IOMM-Lee cells upon Doxorubicin or DMSO treatment; (**E**) EdU assay of the cell proliferation ability in IOMM-Lee cells upon Doxorubicin or DMSO treatment. Scale bar: 125 μm; (**F**) Statistical analysis of the EdU-positive cell ratio; (**G**) Wound healing assay showed delayed closure of the wound gap upon Doxorubicin treatment. (**H**) Statistical analysis of the cell migration in the scratch wound healing assays. (**I**) Transwell invasion and migration assay of IOMM-Lee cells upon Doxorubicin or DMSO treatment. (**J**) Statistical analysis of the cell numbers passing through the transwell chamber. All data were the means ± SD. Results triplicate and repeated at least three times. Results are expressed as mean ± SEM. *P < 0.05, **P < 0.01, ***P < 0.001.

All differentially up-expressed genes were used to the CMap analysis. The top 50 compounds that potentially be the candidates were illustrated in Fig. 5A, alongside with 31 mechanisms by the mode-of-action (MOA) analysis. The result revealed that the top three MOA were the topoisomerase inhibitor, CDK inhibitor, and EGFR inhibitor and the ellipticine, SN-38, etoposide, camptothecin, teniposide, amonafide, and amsacrine shared the topoisomerase inhibitor mechanism from the MOA analysis.

To investigate the efficacy of Doxorubicin (Fig. 5B) for the treatment of meningioma, we assessed the half-maximal inhibitory concentration (IC_50_) of Doxorubicin using CCK-8 assay in IOMM-Lee cell line (Fig. 5C). IC_50_ value of Doxorubicin was 173.3 nM. To assess cell growth over time, cells were treated with Doxorubicin at IC_50_ and the proliferation rates were counted (Fig. 5D). Result showed that the proliferation of IOMM-Lee cells was significantly inhibited compared with that in the DMSO group in time-dependent manner. Moreover, EdU assay revealed that Doxorubicin suppressed IOMM-Lee cell proliferation (Fig. 5E,F). In addition, scratch wound healing assays and transwell assays showed that Doxorubicin suppressed cell invasion and migration (Fig. 5G–J). Taken together, these results suggested that doxorubicin inhibits the proliferation, migration, and invasion of IOMM-Lee cells.

## Discussion

In the study, miR-3605-5p, miR-664b-5p, PNRC2, BTBD8, SLFN13, DGKD, NSD2, EXTL2, and BVES were closely corrected with the malignant progression of meningioma. Previous study has shown that miR-3605-5p could modulate SCABR2 but the finding did not be proved in the laboratory^39^. lncRNA OTUD6B-AS1 could upregulate PNRC2 to inhibit the cellular processes in colorectal cancer^40^. NSD2 has been proved a critical molecule in proliferation, metastasis, and the active transcription of a series of genes^41^. Additionally, NSD2 can also directly methylate PTEN and enhance the DNA damage repair ability in colorectal cancer, and enhance the resistance of cancer cells to chemotherapy^42^. Above that, EXTL2 was corrected with the benign meningioma group and better outcome, which was similar with our results. Mutations in EXT2 could cause the human disorder hereditary multiple osteochondroma, an autosomal dominant disorder characterized by bone deformities and cartilage-capped bony outgrowths, osteochondromas, at the ends of the long bones^43^. BVES was corrected with the non-invasion and the low WHO grade group meningioma. BVES loss was also increase beat-catenin protein levels, leads to Wnt pathway activation and coordinates with Wnt ligand to further increase Wnt signaling expression^44^. Additionally, over-expression of SLFN13 and DGKD confers poor prognosis to the lung cancer patients and the ovarian cancer^45,46^.

The chemotherapy methods of malignant meningioma were limited. There are some reported clinical trials, but the effect was unsatisfactory^8,47^. We used the CMap website to select compound drugs that may have effect on meningioma with specificity^48^. Topoisomerase inhibitor was the most important mode of action. The stimulation of DNA topoisomerase activity could enhance chromosomal segregation by RNA polymerase II clearance during elongation^49^, and chromosomal segregation was also revealed in the GO analysis. Doxorubicin could cause DNA and RNA damage through inhibiting the ongoing of the topoisomerase II, thus induce the cell apoptosis^50^. Doxorubicin is an anthracycline derived from the Streptomyces yeast. Additionally, we found topoisomerase has been proved a high expression in the recurrent meningioma^51,52^. Doxorubicin could inhibit proliferation, migration, and invasion of IOMM-Lee cells in this study. Compared with this, there were cases that revealed that doxorubicin may be a treatment of malignant meningioma^53–56^. And, similar with our results, a recent study also revealed that Doxorubicin was a useful compound for meningioma and the authors also found that ixabepilone was better than Doxorubicin^56^. However, whether ixabepilone was better than Doxorubicin needs the clinical trial to validate the result. The potential compound may ultimately help to find the way of differentiation therapies for meningioma.

Although the prognostic factors and doxorubicin were revealed in this study for meningioma. The underlying mechanisms that contributing to the malignant progression of meningioma was still unclear. GO analysis revealed that chromosome segregation, chromosomal region, and DNA helicase activity were involved in the development of meningioma. About DNA helicase activity, M Mendiola et al. found that hRAD54,which is related to a family of genes involved in DNA helicase activity, is mutated in the sporadic meningioma^57^. Apart from that, cell cycle and systemic lupus erythematosus were revealed in the KEGG analysis. Cell cycle process has been proved that it has an important role in the development of meningioma^58^. And, some reports showed that systemic lupus erythematosus and meningioma happened in a patient^59,60^, which may reveal that progression of meningioma may involve in the immune process and reaction. Next, the GSEA analysis proved that and identified some immune related pathways including activating and supressing. And, Carmen Rapp et al. identified higher numbers of tumor infiltrating T lymphocytes is the biomarker for better outcomes of meningioma^61^. Apart from that, there are also some cancer pathways involving in the malignant progression of meningioma. For instance, Beauchamp et.al revealed that mTORC1/2 inhibition could downregulate NRG1-ERBB3, which increase treatment of NF2-deficient meningioma^62^. Development of malignant meningioma involves a complicated regulatory network and needs more study in the future.

This study presents nine prognostic factors and the effect of Doxorubicin in meningioma. However, the underlying mechanism was still unclear and the prognostic factors should be further studied to assess the prognostic performance. Whether Doxorubicin could be as the potential therapeutic targets in malignant meningioma is warranted in the future studies.

## Supplementary Information

Supplementary Information.

## Data Availability

The datasets analyzed in the study can be found in the supplementary files. And, the RNA-seq data was deposited as fastq format with BioProject name was PRJNA772033. More requests to the clinical information or the information to any data used in the paper can be directed to hxl950513@126.com.
