# Supplementary material for: Prognostic factors and Doxorubicin involved in malignant progression of meningioma
Source: Sci Rep. 2023 Apr 6;13:5632. doi: 10.1038/s41598-023-28996-0 (PMC10079659; doi:10.1038/s41598-023-28996-0)
Supplement: Supplementary file 1 — Supplementary Information. [file 41598_2023_28996_MOESM1_ESM.docx]

**Prognostic Factors and Doxorubicin involved in Malignant Progression of Meningioma**

Xulei Huo^†1234^, Lairong Song^†1234^, Ke Wang^1234^, Hongyi Wang^1234^, Da Li^1234^, Huan Li^1234^, Wei Wang^5^, Yali Wang^6^, Lei Chen^5^, Zongmao Zhao^#7^, Liang Wang^#1234^, and Zhen Wu^#1234^

**Affiliation:** ^1^Department of Neurosurgery, Beijing Tiantan Hospital, Capital Medical University, Beijing, China. ^2^ China National Clinical Research Center for Neurological Diseases, Beijing, China. ^3^Center of Brain Tumor, Beijing Institute for Brain Disorders, Beijing, China. ^4^Beijing Key Laboratory of Brain Tumor, Beijing, China.^5^Department of Neurosurgery, Tianjin Fifth Center Hospital. ^6^Department of Neuro-oncology，Cancer Center，Beijing Tiantan Hospital，Capital Medical University. ^7^Department of Neurosurgery, The Second Hospital of Hebe Medical University.

^†^ These two authors contributed to this article equally.

**Corresponding authors:** Zhen Wu, email: [wuzhen1966@aliyun.com](mailto:wuzhen1966@aliyun.com), Department of Neurosurgery, Beijing Tiantan Hospital, Capital Medical University, Nansihuanxilu 119, Fengtai District, Beijing, 100070, China; Liang Wang, [saintage7@126.com](mailto:saintage7@126.com); and Zongmao Zhao, zzm69@163.com.

**Conflict of Interest:** All authors indicated no potential conflicts of interest.

**Supplementary Figures**

**
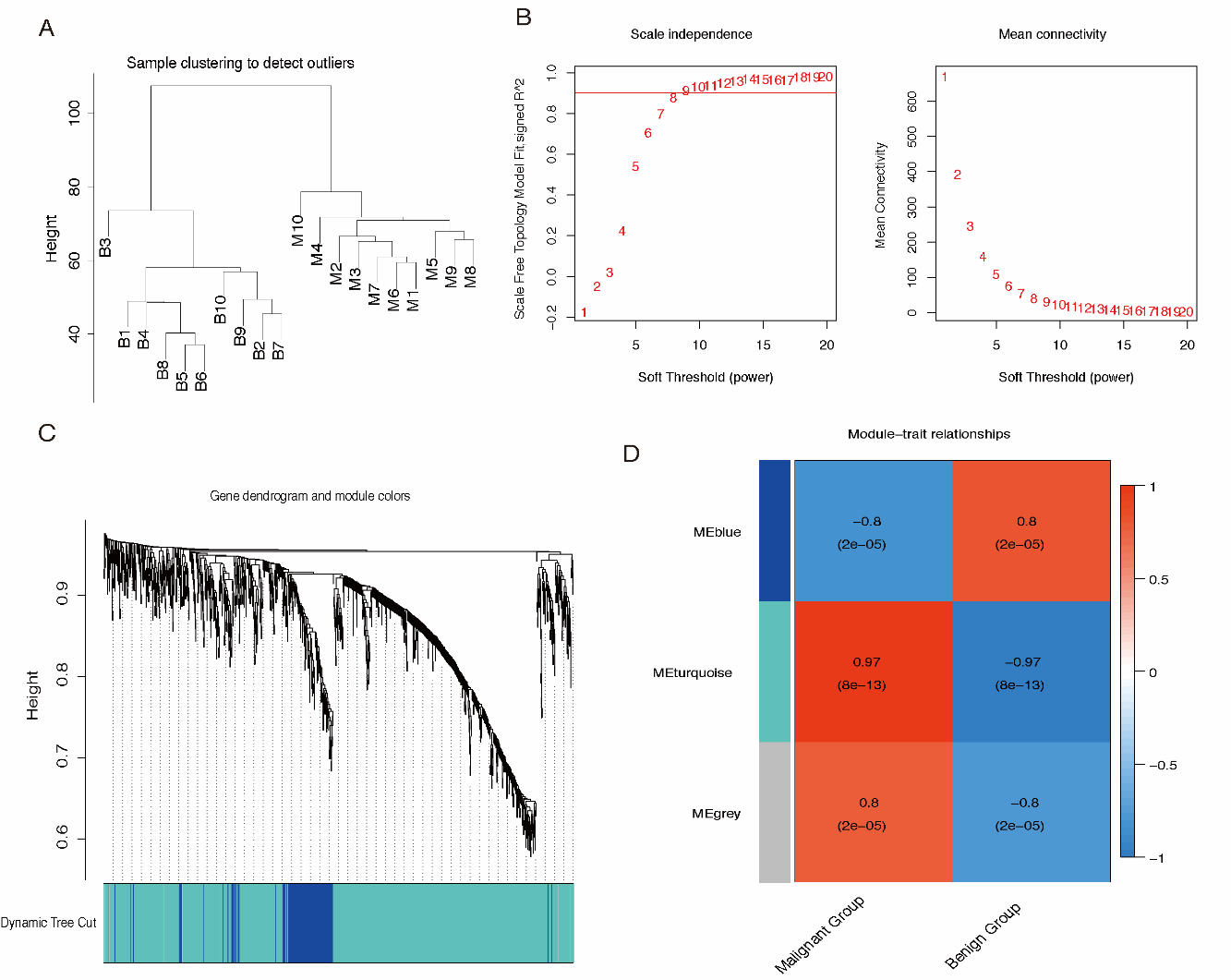
**

**Supplementary Fig. S1 ∣** WGCNA analysis of the differential expressed mRNAs between malignant group sample and benign group samples. (**A**) Test grouping to recognize exceptions dependent on mRNA expression data. Hierarchical clustering analysis was applied to check the heterogeneity of samples and the benign and malignant groups were clustered respectively; (**B**) The relationship between various soft‑threshold power and scale free topology model fit or mean connectivity. When the soft threshold power β reached 8, the scale free topology model fit index was higher than 0.90 and mean connectivity was infinitely approaching 0; (**C**) Five clusters were shown in different colors. Hierarchical clustering analysis was then performed to obtain the weighted co-expression network; (**D**) module‑trait relationship, each row corresponds to a module and column to a trait. In each cell, the connection coefficient (R) was at the upper position while *P*‑value was at the lower position. The heatmap with correlation coefficient (R) and significant difference (*P*-value) revealed the correlation between modules with clinical information.

**
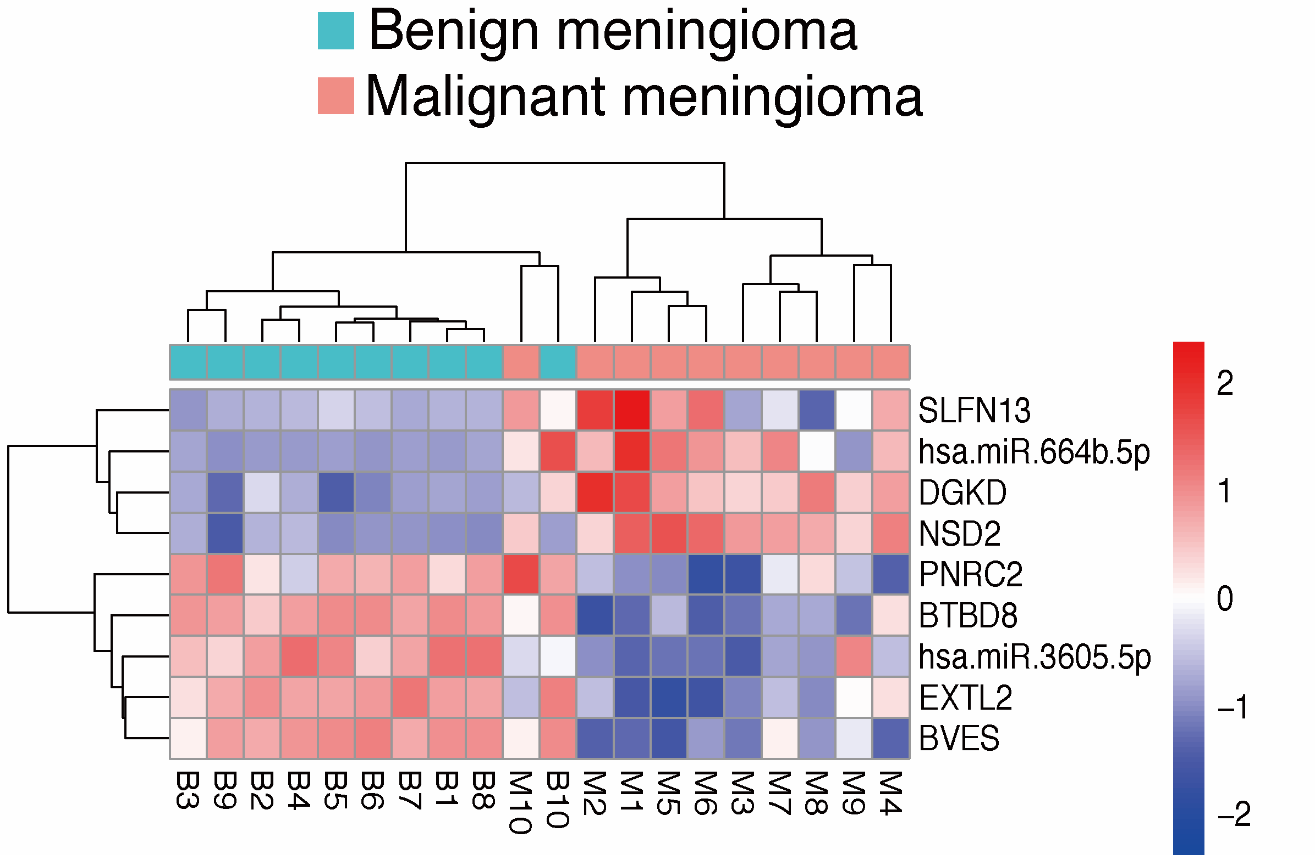
**

**Supplementary Fig. S2 ∣** Heatmap plot of the MPRGs-based prognostic factors in the malignant group and benign group. “ComplexHeatmap” R package (version 3.15) was used to graph the result with R (version 4.1.2, R: The R Project for Statistical Computing).

.

**
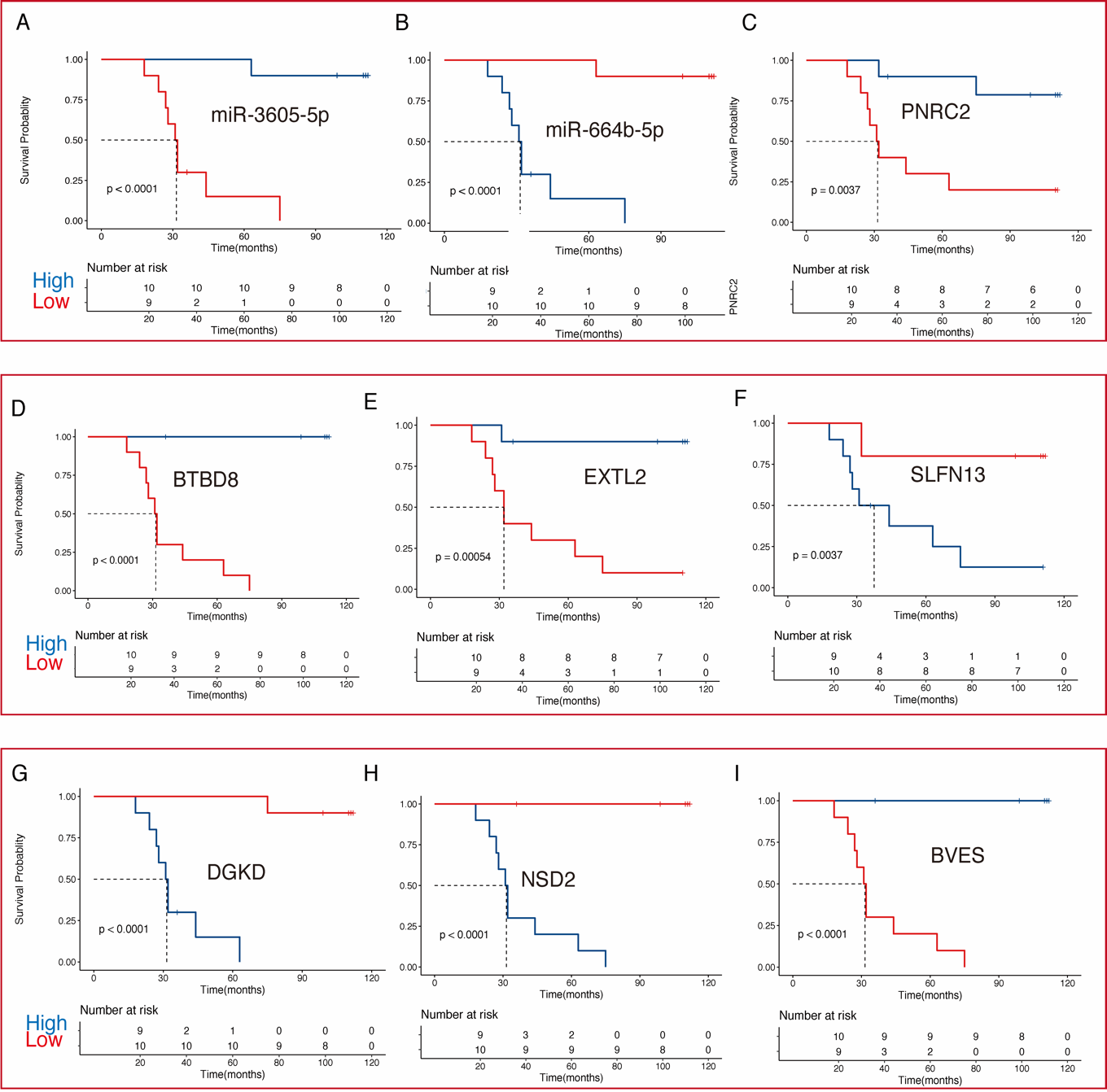
**

**Supplementary Fig. S3∣**Kaplan-Meier survival analysis of the nine MPRGs-based prognostic factors. Meningioma patients with low miR-3605-5p (*P*<0.0001, **A**), PNRC2 (*P<*0.001, **C**), BTBD8 (*P*<0.0001, **D**), EXTL2 (*P*<0.001, **E**), and BVES (*P*<0.0001, **I**) expression level have shorter overall survival time than contrast group patients; Meningioma patients with high miR-664b-5p (*P*<0.0001, **B**), SLFN13 (*P*<0.01, **F**), DGKD (*P*<0.0001, **G**), and NSD2 (*P*<0.0001, **H**) expression level have shorter overall survival time than contrast group patients. Log-rank test was used as the method.

| **Genes** | **Forward Primer** | **Reverse Primer** |
| --- | --- | --- |
| GAPDH | 5′-AGATCCCTCCAAAATCAAGTGG-3′ | 5′-GGCAGAGATGATGACCCTTTT-3′; |
| SLFN13 | 5′-AAGAGAGGGCGAGAGTTATACG-3′ | 5′-GGACGCTCATCCCTGTTGG-3′ |
| PNCR2 | 5′-CAGGCCATGCAAAATGGGG-3′ | 5′-ACCTGGGACCTGATAAGCTAGA -3′ |
| EXTL2 | 5′- CTTCCTGGGAGAGTAATGGGG-3 | 5′-CCTACGCAACATGAGCATCTTG-3′ |
| NSD2 | 5′-ACCGCGAGTGTTCTGTGTTC-3′ | 5′-ACCGCGAGTGTTCTGTGTTC-3′ |
| DGKD | 5′-CTTCGAGGGCGAACGCTTTA-3′ | 5′-TTTTGGTACTGGATTCAGCTACG-3′ |

**Supplementary Tables**

**Table S1. The Primers of mRNAs in the qRT-PCR experiment**

| **Genes** | **Forward Primer** |
| --- | --- |
| U6 | 5′-CTCgCTTCggCAgCACA-3′ |
| hsa-miR-3605-5p | 5′-TgAggATggATAgCAAggAAgC-3′ |
| hsa-miR-664b-5p | 5′-TgggCTAAgggAgATgATTggg-3′ |

**Table S2. The Primers of miRNAs in the qRT-PCR experiment**
